# Devotion for newborn children

**Published:** 2016

**Authors:** VL Purcarea

**Affiliations:** * "Carol Davila" University of Medicine and Pharmacy, Bucharest, Romania

“ROMMEDICA - Medical Instruments and Equipment International Fair –” took place at Romexpo Exhibition Center in Bucharest between the 14th and the 16th of April 2016. 

The event, which represented an important source of information for the representatives of the health system in the public and private sector, was and still is the most important annual meeting place for the specialists in medicine and pharmacy, welcoming the doctors with a rich offer of technology and medical devices for the facilitation and improvement of the medical act.

The exhibition presented the latest trends in the medical field, being an event addressed both to the companies that produce medical devices and software, pharmaceutical companies, banks, financing and leasing companies, private medical centers and medical associations, as well as to the current academic and professional publications. 

During the event, conferences and seminaries moderated by important personalities in the medical field have been organized, and products, technologies, information, and specialty publications have also been launched. 

**Fig. 1:**
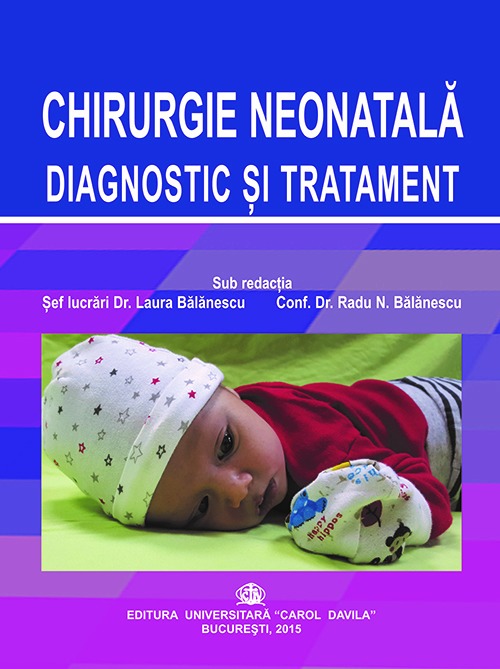
Treatise of “Neonatal surgery, diagnosis, and treatment”

Regarding the latter, like every year, at “Carol Davila” University Press stand, useful and valuable medicine treaties, which raised a great interest among the readers, were presented. “Neonatal surgery, diagnosis, and treatment” can be mentioned among the most important medical treaties presented. It is a neonatal treatise that was missing from the medical literature in Romania, being realized under the editorial coordination of **Prof. Radu Bălănescu, MD, PhD and Assoc. Prof. Laura Bălănescu, MD, PhD**.

The treatise contains all congenital diseses which can endanger the life of newborn children, presenting the experience and the constant efforts that have been made for 25 years of medical activity of an important team of enthusiastic and experienced pediatic surgeons, who have tried and are still trying, through their day to day activity, to help their little patients to win the fight for life. 

**Fig. 2:**
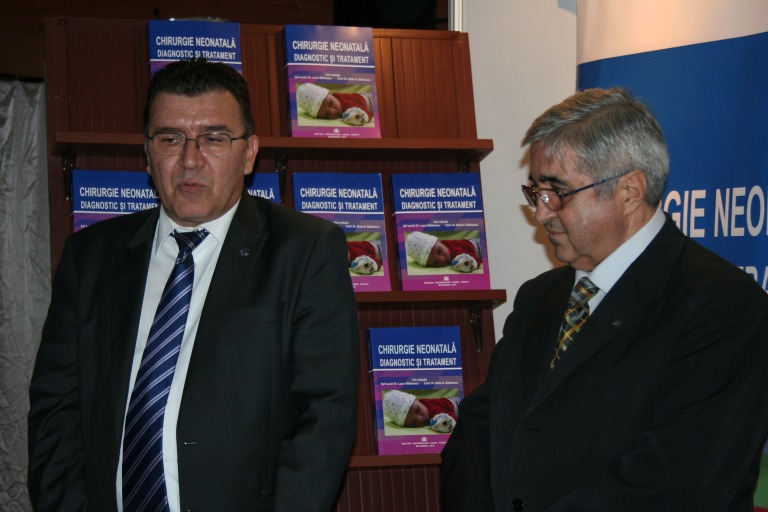
Prof. Radu Bălănescu, MD, PhD and Prof. Dr. Eng. Victor Lorin Purcărea, Manager of “Carol Davila” University Press

The remarkable manuscript contains **552** pages, **100** figures and **30** tables, being structured in **5** parts and **43** chapers which present very important subjects such as the antenatal diagnosis of congenital malformations; obstetrical traumatic pathology of newborn child; pilor hypertrophic stenosis and pilor congenital malformations; kidney congenital malformations; kidney neonatal tumors; etc. 

**Fig. 3:**
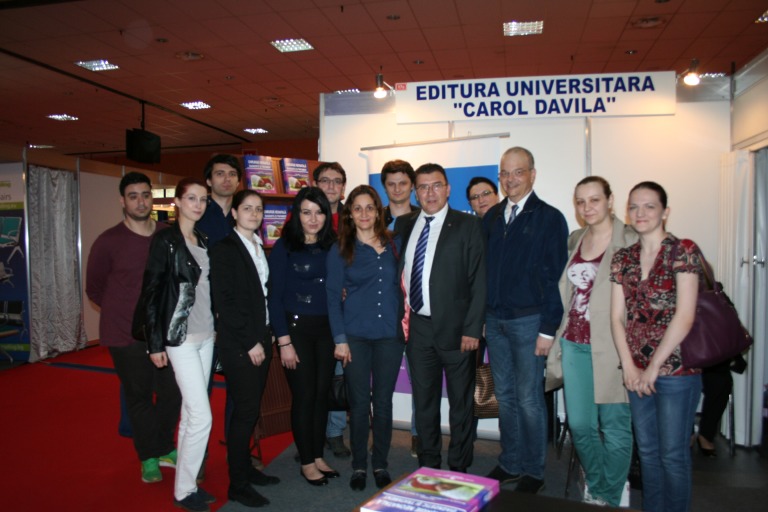
Coordinator editors Prof. Radu Bălănescu, MD, PhD and Assoc. Prof. Laura Bălănescu, MD, PhD, surrounded by friends - authors and colleagues

The authors state the following in the preface of the treatise: *„we have tried and hopefully managed to deal with all the congenital diseases which can endanger the life of the newborn children, both as immediate emergencies and as late emergencies... We consider that time has finally come to have the possibility to treat these patients in the same way, all over Romania, respecting the same medical protocols applied by our colleagues around the world. ....We hope that the usefullness of this treatise will be appreciated both by the doctors who work in famous medical clinics and by the ones who have to establish a diagnosis or to treat some cases without having the support of a great and skilled team in this field... Together with our other dedicated colleagues in Bucharest and in the country, we wish and we are trying to complete the training by organizing specialty courses and inviting speakers from other clinics in Europe, with the final goal of obtaining the best results regarding the treatment of the children patients, which the medical system in Romania deals with...”*

**Executive Editor** **Prof. Dr. Eng. Victor Lorin Purcarea**

